# The miRNomics of antiretroviral therapy-induced obesity

**DOI:** 10.1007/s10142-025-01585-2

**Published:** 2025-04-05

**Authors:** Niska Majumdar, Bishwa R. Pokharel, Abigail Dickerson, Andreea Cruceanu, Smit Rajput, Lok R. Pokhrel, Paul P. Cook, Shaw M. Akula

**Affiliations:** 1 Department of Microbiology & Immunology, Brody School of Medicine at East Carolina University, Greenville, NC 27834 USA; 2 Department of Internal Medicine, Brody School of Medicine at East Carolina University, Greenville, NC 27834 USA; 3 Department of Public Health, Brody School of Medicine at East Carolina University, Greenville, NC 27834 USA

**Keywords:** HIV, Obesity, MiRNA, HAART, ART

## Abstract

Human immunodeficiency virus (HIV) is a retrovirus that incorporates its genetic material into the host’s chromosome. The resulting diseases and related conditions constitute a global health problem as there are no treatments to eliminate HIV from an infected individual. However, the potent, complex, and active antiretroviral therapy (ART) strategies have been able to successfully inhibit HIV replication in patients. Unfortunately, obesity following ART is frequent among HIV-infected patients. The mechanism underlying ART-induced obesity is characterized based on expression of traditional markers such as genes and proteins. However, little is known about, yet another key component of molecular biology known as microRNAs (miRNAs). Micro-RNAs are ~ 22 base-long non-coding nucleotides capable of regulating more than 60% of all human protein-coding genes. The interest in miRNA molecules is increasing and their roles in HIV and obesity are beginning to be apparent. In this review, we provide an overview of HIV and its associated diseases, ART-induced obesity, and discuss the roles and plausible benefits of miRNAs in regulating obesity genes in HIV-infected patients. Understanding the roles of miRNAs in ART-induced obesity will aid in tracking the disease progression and designing beneficial therapeutic approaches.

## Introduction

Human immunodeficiency virus, or HIV is a retrovirus that affects the immune system (Marra et al. 2024). HIV can lead to a host of medical ailments bolstered by a weak immune system (Scott and Worku 2024). The main ailment being Acquired Immunodeficiency Syndrome (AIDS), that gives rise to a variety of other opportunistic infections (OIs) (Admasu et al. 2024). Treatment therapies like antiretroviral therapy (ART) or highly active antiretroviral therapy (HAART), have been successful in prolonging the lives of several individuals with HIV and OIs that follow. However, due to its chronic nature, the treatments and clinical responsibilities have a worsening impact on the patients. Thus, HIV and AIDS cause an increase in debt for those infected and their families, including a reduction in investment towards daily lifestyle standards such as nutritional foods, education, occupational exceptions, and business. Social impact also impedes individuals globally from proper access to HIV diagnoses and treatments. Public stigma often influences whether individuals seek to get tested. This is often due to judgements about promiscuity, perceived lack of responsibility and hygiene, or notions of drug abuse (Chayama et al. 2024; Lin et al. 2024; Nice et al. 2024).

The first approved antiretroviral (ARV) drug used to treat HIV infections was zidovudine (AZT) in 1987 (Tseng et al. 2015). Since then, there have been over 30 different drugs developed to combat HIV infections. HIV treatment has presented many pharmacological challenges. Despite these impediments, we have achieved outstanding results over the last four decades in terms of ARVs access and coverage; primarily due to the introduction of generic medications worldwide (Ripamonti and Leon 2024). Obesity and bone fracture risk are the primary comorbidities frequently linked to long term antiretroviral therapy (ART) use in HIV patients.

MicroRNAs (miRNAs) are one of the many types of noncoding RNA found in eukaryotes that regulate a variety of cellular and viral genes. In fact, these short single strands of nucleotides are predicted to modulate the majority of all human protein coding genes. In a recently published study from our lab, we have detailed the biogenesis and roles of these miRNAs in viral pathogenesis (Bauer et al. 2023). This review will discuss HIV, HIV-induced diseases, its global impact, ARTs, ART-induced obesity, roles of miRNAs in HIV infections and ART-induced obesity, and a concluding remark on how we can exploit miRNAs as diagnostic biomarkers and develop next generation therapeutics.

### Virus

HIV is classified as a lentivirus and can be of two types, HIV 1 and 2 (Santana et al. 2023; Sharp and Hahn 2011). Lentiviruses are the subclass genus of the family *Retroviridae*, HIV-1 and −2 being primate lentiviruses. Lentiviruses are lifelong offenders due to their ability to mutate at record rates. They integrate into the hosts’ chromosome and are not affected by hosts’ immune system (Engelman and Kvaratskhelia 2022; Suleman et al. 2022). If left untreated, HIV can cause AIDS. There is currently no effective treatment for HIV. There are therapeutic regimens that help curb the effects of HIV to increase the longevity of patients and protect partners from contracting the virus. The primary mode of transmission in humans is through sexual contact via the lower genital and rectal mucosa. Other mechanisms of transmission include blood, placenta, and gastrointestinal mucosa (Hladik and McElrath 2008; Yoshimura 2017).

### Disease associated with HIV

Being a virus that affects the immune system, HIV can lead to several medical ailments boosted by host’s weakened immune system. AIDS is the primary disease associated with HIV. AIDS is the most advanced stage of HIV infection. Individuals receive an AIDS diagnosis when they develop certain OIs, or their CD4 + T cell (or T4 cells) count drops below 200 cells per milliliter of blood (Carinelli et al. 2015). OIs are more frequent and severe for HIV patients due to the compromised immune system (Sutini et al. 2022). Diseases associated with HIV based on the ranges of CD4 + counts is presented in Table 1. Some others include weight loss, neurocognitive disorders, wasting syndrome, isosporiasis, tuberculosis, and anal cancer (Anderson et al. 2021; Iordanov et al. 2021; Korn et al. 2022; Pettit et al. 2023). OIs are currently less common due to modern treatment regimens such as antiretroviral therapy (ART) but are still possible to develop overtime in hosts who are unaware of having HIV, those refusing treatments, or those undergoing ineffective treatments. Often, people who develop OIs due to HIV fail to get diagnosed for the virus and are either sent away or treated for only the symptoms of the OI. People living with HIV (PLWH) contract OIs when their infection-fighting CD4 + cells fall below 200 cell/mm^3^ and in some cases, under 500 cell/mm^3^ (Anglaret et al. 2012; Birmeka 2023; Ramesh et al. 2015). Apart from general OIs, the virus also affects neurological aspects (Uwishema et al. 2022). Complications such as HIV-associated neurocognitive disorders (HAND) or central nervous system opportunistic infections (CNS-OIs) can also occur (Elendu et al. 2023; Oprea et al. 2023).

**Table 1:** Diseases associated with HIV based on CD4 + counts

Cell count	Associated OIs
< 250	Tuberculosis (Auld et al. 2020)
	Coccidioidomycosis (Ampel et al. 1993; Blair et al. 2019)
< 200	Herpes simplex viruses (HSV) (Schiffer et al. 2016)
	Microsporidiosis (Al-Brhami et al. 2022)
	Mucocutaneous candidiasis (Anwar et al. 2012)
	Cryptococcus neoformans (Mimicos et al. 2022)
	Pneumocystis jirovecii pneumonia (PCP) (Lin et al. 2016)
< 150	Histoplasma capsulatum (Almeida et al. 2019)
< 100	Cryptosporidiosis (Izadi et al. 2012; Wang et al. 2018)
	Toxoplasma gondii encephalitis (Li et al. 2020b; Mamfaluti et al. 2023)
	JC virus infection (Abrao et al. 2020)
< 50	Cytomegalovirus (Ali et al. 2023)
	Mycobacterium avium complex (MAC) (Yuan et al. 2023)
	Cerebral toxoplasmosis (Pekova et al. 2021)
	Bartonellosis (Koehler et al. 2003; Secamilli et al. 2024)
Other co-infections	Syphilis (Fan et al. 2021)
	Human papillomavirus infection (HPV) (Cambrea et al. 2022)
	Hepatitis B (Anderson et al. 2024; Kamurai et al. 2024)
	Hepatitis C (Adekunle et al. 2022)

### HIV therapeutics

The ART journey began in 1987 with the introduction of nucleoside reverse transcriptase inhibitor (NRTI) and zidovudine (ZDV) (Volberding et al. 1995). This was the first ARV class of approved drugs by the FDA. Subsequently, protease inhibitors (PIs in 1995) and non-nucleoside reverse transcriptase inhibitors (NNRTIs in 1996) were introduced as treatment strategies (Weber et al. 2021; Zhuang et al. 2020). HAART was launched initially as a cocktail of two NRTIs, and a PI/NNRTI, with an increased potency in suppression. This led to a significant reduction in mortality rate among individuals with HIV (Tseng et al. 2015). The production and efficacy of these cocktail type drugs lead to the development of combination therapies (cART). Now, we have several other combinations using NRTIs as a backbone. Since the discovery of HIV/AIDS, the FDA has approved 33 ART drugs (Toledo et al. 2023). The current FDA approved drug classifications for HIV treatment are listed in Table 2.

**Table 2:** Current FDA approved drug classifications for HIV treatment

Drug Class	Current Drug(s)	Mechanism of Action
CCR5 antagonists	Maraviroc	A type of entry inhibitor. CCR5 inhibitors prevent the binding of viral envelope glycoprotein (gp120) to CD4 + cells and the chemokine co-receptor CCR5 (Zhou et al. 2024)
Post-attachment inhibitors	Ibalizumab	A type of entry inhibitor. Post-attachment inhibitos are monoclonal antibodies that non-competitively target CD4 + cells to prevent the necessary conformational changes in the CD4 + -gp120 complex that facilitate viral entry (DeJesus et al. 2024; Rizza et al. 2019)
Fusion inhibitors	Enfuvirtide (T-20)	An entry inhibitor. Fusion inhibitors like T-20 can specifically bind to gp41, and disrupt the gp41-mediated fusion process between the viral and host cell membranes, thereby inhibiting the critical step of viral entry into the host cell and preventing subsequent HIV infection (He et al. 2024)
Nucleoside reverse transcriptase inhibitors (NRTIs)	Emtricitabine Tenofovir-alafenamide	NRTIs are active after metabolism, hence are considered as prodrugs. They block viral DNA synthesis. In active form, they generate ddNTPs and ddNDPs, which compete with dNTPs for substrate binding by the viral reverse transcriptase enzyme. This allows for early termination of viral RNA transcription (Holec et al. 2017; Saehlee et al. 2024)
Non-nucleoside reverse transcriptase inhibitors (NNRTIs)	Rilpivirine Etravirine	NNRTIs can competitively bind near the catalytic region of RT and block the RT initiation complex during early viral transcription. Thus, preventing viral transcription. NNRTI targets a different binding pocket of the RT enzyme compared to NRTI (Wang et al. 2024b)
Protease inhibitors (PIs)	Atazanavir Darunavir	PIs target and disable viral aspartyl protease enzyme activity by cleaving the immature polyprotein and preventing formation of mature proteins necessary for viral replication (Alkashef and Seleem 2024)
Integrase strand transfer inhibitors (INSTIs)	Cabotegravir Dolutegravir	Viral integration is catalyzed by the virally encoded integrase protein within a nucleoprotein assembly called an intasome. The conserved intasome core holds the viral DNA ends and has two active sites for integration. INSTIs target these sites, as well as the processed vDNA ends. Once bound, they stop the virus from attaching its DNA to the host (Hikichi et al. 2024; Jozwik et al. 2020)
Capsid inhibitor	Lenacapavir Islatravir	While the mechanism of each kind of capsid inhibitor varies, they all target the viral capsid. Lenacapavir for instance, disrupts multiple stages of the HIV life cycle by binding to two capsid protein subunits, hindering crucial interactions for nuclear import, virion assembly, and capsid formation. Viruses produced with lenacapavir have defective capsids, allowing entry into cells but preventing replication (Bester et al. 2020; Diamond et al. 2024)
PK enhancers	Cobicistat	A CYP3A inhibitor. Prevents ARVs from being metabolized and lost too early before desired antiretroviral effects can be established (von Hentig 2016). Each kind of PK enhancer has a different mechanism of action

*ddNTP* Dideoxynucleotide triphosphate, *ddNDP* Dideoxynucleotide diphosphate, *dNTP* Deoxynucleotide triphosphate, *RT* reverse transcriptase, *CCR5* C–C chemokine receptor type 5, *PK* Pharmacokinetic

Apart from drugs specifically for HIV positive individuals, uninfected partners were offered preventative treatment called Pre-Exposure Prophylaxis (PrEP) (Felix Sanni et al. 2024). This regimen consists of daily doses of tenofovir and emitricitabine (both NRTI antiretrovirals) combined in a single pill (Sullivan et al. 2024). The HIV therapeutic strategies are continuously evolving for the better.

### The effects of Antiretrovirals (ARVs) on obesity

Any weight gain after beginning the ARV treatment regimen is considered to reduce mortality rates in individuals who are underweight or normal weight (Yuh et al. 2015). But with excess adiposity due to potential metabolic dysregulation, other risks arise. Fatty liver disease, diabetes mellitus, cardiovascular disease, and neurocognitive impairment are considered some of the hallmarks of ARV intake (Iacob and Iacob 2022; Letendre et al. 2023; Nolan et al. 2021; Vos and Venter 2021). In a multi-cohort study, comparing HIV positive individuals from the North America AIDS Cohort Collaboration on Research and Design (NA-ACCORD) and United States National Health and Nutrition Examination Survey (NHANES), data showed a rise in obesity after initiating therapy (Koethe et al. 2016). More than 14,000 HIV positive individuals were studied and an overall increase in median BMI by ~ 4% and the percentage of obese individuals increased from 9 to 18% upon ART initiation (Koethe et al. 2016).

**Fig. 1:**
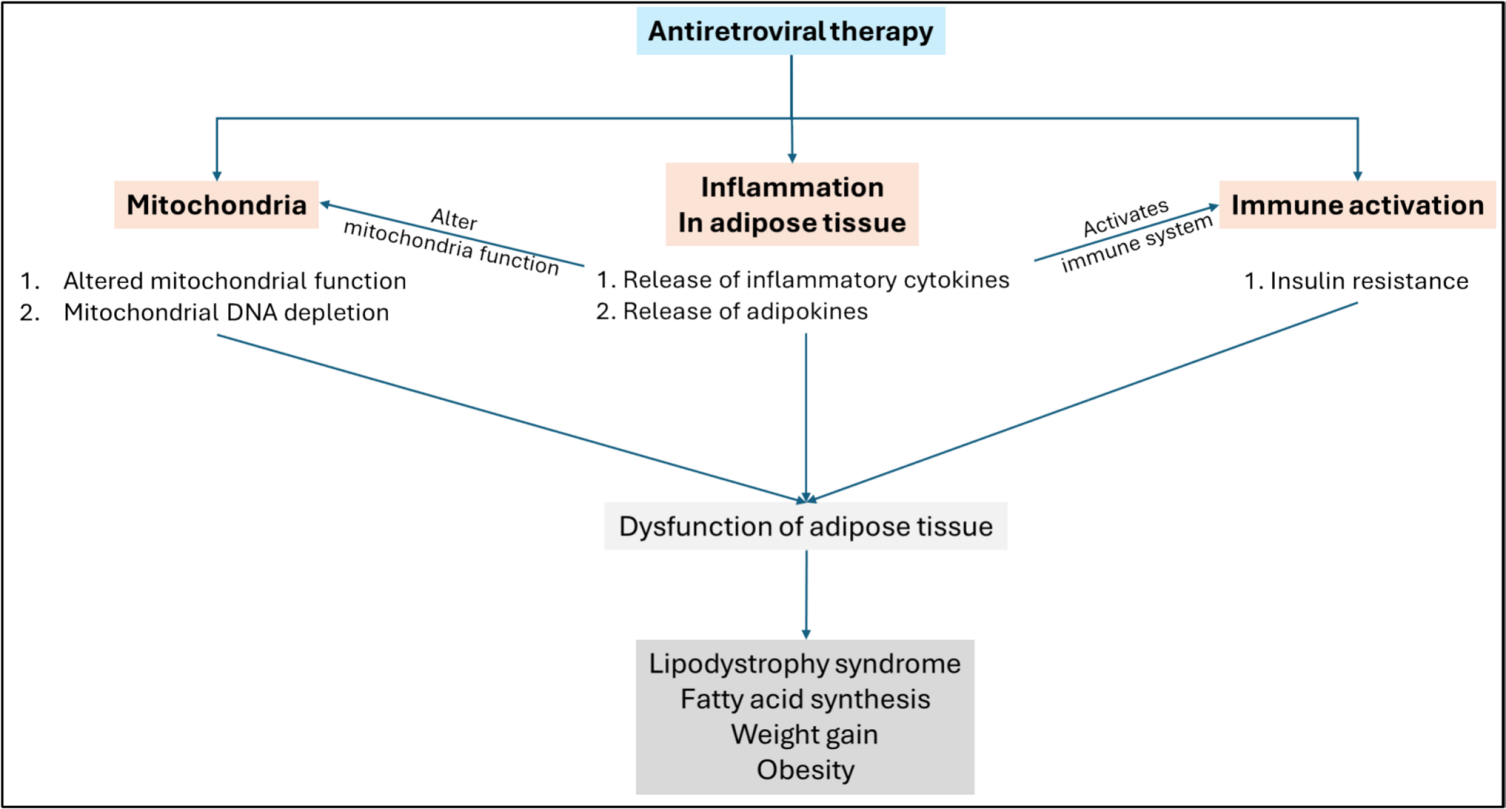
ARVs may induce obesity via modulating mitochondrial function (Rodriguez et al. 2024), inflammation (Mave et al. 2016), or activation of immune system (Pedro et al. 2018)

Dyslipidemia, inflammation, and insulin resistance are the major adverse effects of the ARV regimen, often leading to complications with weight gain, and obesity (Jao et al. 2024; Mafumhe et al. 2024; Mohan et al. 2023; Wang et al. 2024a). The three major mechanisms by which ARV treatments may drive obesity are outlined in Fig. 1. Recent studies have shown specific types of ART drugs, such as INSTI and NRTI combination drugs, have been considered as contributors to weight gain. Additionally, INSTIs may interfere with the melanocortin signaling system, leading to appetite stimulation (Kousari et al. 2024; Wood and Huhn 2021; Zhao et al. 2023). Another study was able to surmise that higher dolutegravir (DTG) hair concentrations were linked to body weight gain in nonobese women, suggesting a potential dose-dependent effect on metabolism, adipocyte function, or appetite (Lahiri et al. 2023). Even with all these findings, the underlying mechanism of action is yet to be understood. Newer regimens tend to show more signs of weight gain, with INSTIs showing higher rates. Studies suggest Dolutegravir (INSTI) and bictegravir (INSTI) may lead to greater weight gain than elvitegravir (INSTI) /cobicistat (PK enhancer) (Murray et al. 2023; Sax et al. 2020). Changes in daily average dietary intake have also been shown to aid in excessive weight gain in people with HIV (Bailin et al. 2024; Hernandez et al. 2017; Pepin et al. 2018). Compared to the average dietary standards among healthy individuals, HIV-positive individuals do not tend to meet the U.S. government-recommended nutritional standards.

### Obesity and regulatory genes

Obesity is a disease caused by excessive weight gain (Delfan et al. 2024). In 2000, WHO classified obesity based on BMI. A normal BMI is considered 18.5–24.9, while for obesity, it is categorized as 25–29.9 (type I), 30–39.9 (type II), and over 40 (type III) (Paolacci et al. 2019). A new term, “Diabesity” has been on the rise that helps understand the bidirectional relationship of obesity and diabetes (Babicki 2024). Obesity is a global issue that categorizes several other comorbidities ranging from diabetes, heart disease, and cancer (Abrao et al. 2020; Jiang et al. 2024; Klein et al. 2022; Pati et al. 2023). A detailed review on classification and epidemiology of obesity was published and last updated by Purnell in 2025 (Purnell 2000).

Apart from several other factors (WHO report; March 2024), genetics also play a role in biochemical mechanisms that eventually cause obesity. A study confirmed the potential role of macrophage-derived NPY in the development of obesity. Specifically, four-and-a-half LIM-Domain Protein 2 (FHL2)-driven NPY expression in macrophages impact lipid metabolism in adipocytes, partly, by inhibiting the machinery responsible for thermogenic lipolysis (Sommer et al. 2023). This resulted in an obesogenic environment for the body. Genetic variations can also lead to obesity: example, a rare and likely pathogenic MRAP2 variant (potentially deleterious effect in MRAP2) and some POMC variants (2 mutations predicted to impair protein function) existed in a cohort of severely obese Brazilian adults (da Fonseca et al. 2021). Transcription factor single-minded 1 (SIM1), and its mutations are associated with a history of obesity (Gonsalves et al. 2020; Panera et al. 2022). Genetic mutations are a big part of obesity and further research can help discover novel mutations/variants which could act as biomarkers or targets for obesity therapeutics.

Some potential biomarkers for obesity include genes like early growth response 2 (EGR2), gremlin-1 (GREM1), and neuropeptide Y1 receptor (NPY1R). EGR2 is upregulated in adipose tissue of obese subjects in humans (Hua et al. 2023). GREM1 is overexpressed in type 2 diabetes, as well as in non-alcoholic fatty liver disease (Baboota et al. 2022). NPY1R has been associated with overeating and reduced energy expenditure (Zoccali et al. 2021). Genes like caspase-1 (CASP1), insulin growth-factor-binding protein 2 (IGFBP2) and docking protein 6 (DOK6) are significantly overexpressed in the synovial joint tissues of obese individuals (Acharjee et al. 2024). Studies established a direct link between the polymorphisms in leptin (LEP) and adiponectin (ADIPOQ) genes, with excessive weight gain and obesity (Mendez-Hernandez et al. 2017).

In HIV patients, genetic variations can also lead to obesity. Genetic variations in the fat mass and obesity-associated (FTO) gene can be attributed to fatty liver disease in HIV positive individuals (Nunez-Torres et al. 2017). Fatty liver disease is a common symptom of obesity (Charlot et al. 2024; Divella et al. 2019). This could be attributed to the individuals’ lifestyle and eating trends prior to infection and their eating habits after initiation of ART, regardless of how the drug affects metabolic pathways (Taramasso et al. 2024). A polymorphism in transmembrane protein 163 (TMEM163), leading to weight gain was reported upon treatment with dolutegravir (INSTI) and tenofovir alafenamide (NRTI) (Cindi et al. 2021). The longer use of cART and PIs were associated with decreased expression of brown and beige fat genes (UCP1, PGC1α, PRDM16) involved in heat production, potentially contributing to metabolic complications (Koethe et al. 2020; Srinivasa et al. 2019). While there is some evidence suggesting genetic changes upon HIV infection would lead to obesogenic results, most cases of obesity in HIV positive people seem to arise from ARV treatments and lifestyle changes. Finally, miRNAs also play a major role in biochemical and metabolic pathways (lipogenesis, adipogenesis, lipid metabolism) that may cause obesity (Benavides-Aguilar et al. 2023).

### What are microRNAs?

**Fig. 2:**
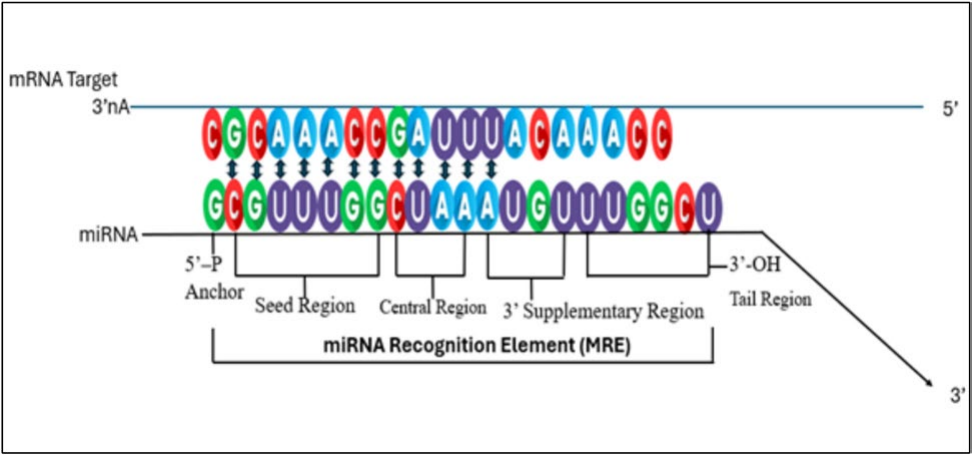
Key regions in miRNA structure. the 5'-P anchor, which is crucial for miRNA stability and its incorporation into the RNA-induced silencing complex (RISC) (Noland et al. 2011); the seed region, typically covering nucleotides 2–7 from the 5' end essential for recognizing and binding complementary sequences on the target mRNA(Kilikevicius et al. 2022; McGeary et al. 2022); the central region which provides additional stability in the miRNA-mRNA interaction; the 3' supplementary region, enhancing binding specificity and stability by complementing sequences downstream on the mRNA (McGeary et al. 2022); and the 3'-OH tail region, aiding in the overall stability of the miRNA within the RISC. The presence of these organized areas allows the miRNA to efficiently direct the RISC to its target mRNA. This may result in either the breakdown of the mRNA or the prevention of its translation, hence controlling gene expression in different biological processes (Iwakawa and Tomari 2022)

MicroRNAs (miRNAs) are small non-coding RNAs, generally about 22 nucleotides in length (Bartel 2004; Ko et al. 2024). The core sections of a typical miRNA can be further defined as depicted in Fig. 2. They originate from hairpin-shaped precursor molecules encoded within the genomes of animals, plants, and viruses. miRNAs can target many distinct messenger RNAs (mRNAs) based on their seed regions and specificity to the UTR of the mRNAs (Bauer et al. 2023; Lee and Shin 2012). Similarly, a specific mRNA may bind to various miRNAs, either at the same time or depending on the environment (Broughton et al. 2016). miRNAs play a crucial role in controlling gene expression and regulating numerous biological processes by inducing translational repression or hastened RNA degradation (Zhang et al. 2021). The miRNA-associated RISC (miRISC) can disrupt the translation process at multiple stages, including initiation, post-initiation, and even elongation (Diener et al. 2024). According to the entries obtained from mirBase dataset (v22), the total number of mature miRNAs detected in humans is 2,654 but some are yet to be functionally validated (Kozomara et al. 2019). The canonical biogenesis of cellular miRNAs during viral infections was recently reviewed by us as a meticulously regulated process involving several critical steps (Bauer et al. 2023). Briefly, it begins with the transcription of primary miRNAs (pri-miRNAs) by RNA polymerase II from dedicated miRNAs’ genes within the genome (Uemura and Ohyama 2024). Pri-miRNAs are lengthy molecules, often spanning several kilobases, and they contain stem-loop structures. These molecules undergo processing in the nucleus by the Microprocessor Complex, which includes the RNase III enzyme Drosha and its crucial cofactor DGCR8. This processing results in the production of precursor miRNAs (pre-miRNAs) (Shang and Lai 2023). Exportin-5 is generally responsible for transporting pre-miRNAs from the nucleus to cytoplasm in a Ran-GTP-dependent manner in most cases (Alquraan and Khabour 2023; Wu et al. 2018). Upon entry into the cytoplasm, the RNase III enzyme Dicer processes these pre-miRNAs into mature miRNA duplexes (Lee et al. 2023; Michlewski and Caceres 2019). Generally, one strand of the miRNA duplex known as the guide strand is incorporated into the RNA-induced silencing complex (RISC), while the other strand, termed the passenger strand is usually degraded (Medley et al. 2021). The mature miRNA within RISC directs the complex to specific mRNA targets, resulting in either mRNA degradation or translation inhibition, thereby playing a crucial role in post-transcriptional gene regulation (Johnson et al. 2024). Additional non-canonical miRNA biogenesis pathways exist and they are broadly classified as Drosha-independent and Dicer-independent pathways (Shang et al. 2023).

Typically, miRNAs bind to the 3′ UTR of target mRNAs to alter gene expression (Ha and Kim 2014; Vaghf et al. 2022). But miRNAs aren’t limited to just the 3’ UTR. miRNAs may also interact with other noncanonical areas such as the 5′ UTR, and protein coding sequences (CDSs) (Abrao et al. 2020; Broughton et al. 2016; Ryczek et al. 2023). Newer research poses to establish that certain miRNAs can also target specific gene promoters to cause transcriptional gene activation or silencing. For example, miR-320 binds to the promoter sequence of POLR3D gene and silence transcription via the Argonaute (Ago) 1 protein (Ago1) (Kim et al. 2008; Sarkar and Kumar 2025). The Ago1 and Ago2 proteins play a key role in the transcription regulation of certain noncanonical miRNAs (Gu et al. 2024).

Finally, miRNAs are also found in exosomes, microvesicles, or high-density lipoprotein particles across a range of biological fluids (Coenen-Stass et al. 2019; Condrat et al. 2020). They are present in all sorts of body fluids ranging from serum, plasma, saliva, sweat, lacrimal fluids, seminal fluids, urine, cerebrospinal fluid, milk, and vaginal secretions (Fujimoto et al. 2019; Kumar and Reddy 2016). Such ubiquitously present miRNAs are being used as a tool to understand various biological processes.

### miRNAs in the biology of obesity, and HIV-associated metabolic disorders

miRNAs have emerged as influential markers in obesity-and-related comorbidities. For instance, miR-129-5p is over-expressed in obese patients and positively correlated with obesity metrics like BMI and fat percentage. miR-129-5p has potential to target ATG7-related autophagy signaling network that regulates white and brown adipogenesis (Fu et al. 2019). miR-33 regulates key steps in reverse cholesterol transport, including high-density lipoprotein (HDL) biogenesis and bile acid synthesis. It also influences fatty acid oxidation, very low-density lipoprotein (VLDL) secretion, and glucose metabolism by controlling the expression of related genes. Hence suppressing hepatic miR-33 could likely reduce obesity-driven liver fibrosis and promote liver regeneration by enhancing fatty acid oxidation and increasing cell cycle regulator expression (Price et al. 2021). Overexpression of miR-29a is now known to suppress PPARδ expression leading to reduced PGC-1α levels, impaired insulin-dependent glucose uptake, and ATP production. It also decreases GLUT4 levels in skeletal muscle, further reducing glucose uptake. These changes contribute to metabolic disease secondary to insulin resistance in skeletal muscles (Zhou et al. 2016). A recent study by Pokharel et al. 2024, confirms the potential of plasma miR-9, miR-29a, miR-192, and miR-375 as biomarkers for early detection of glucometabolic disorders in Nepali adults. miR-29a was the most effective in distinguishing T2DM patients, while miR-192 and miR-375 also showed strong diagnostic value (Pokharel et al. 2024). These miRNAs, such as miR-129-5p, miR-33, and miR-29a, are involved in regulating fat metabolism, cholesterol transport, and glucose uptake, impacting obesity-related metabolic disorders. With new results linking more miRNAs to the outcomes of obesity and weight gain related symptoms, much larger-scale studies are needed to further validate these findings and explore the molecular mechanisms linking these miRNAs to metabolic disorders.

miRNAs are also being documented to serve as potential diagnostic markers for HIV and its associated comorbidities (including obesity and obesity related symptoms). miRNAs found in the serum or peripheral blood mononuclear cells of HIV-1 infected patients can influence the progression of the infection by regulating viral proteins or affecting the host factors associated with HIV-1 replication (Sadri Nahand et al. 2020; Sun et al. 2016). miRNAs have been reported as an early biomarker of HIV-1 infection (Muwonge et al. 2022). A set of six miRNAs correlated with metabolite markers of inflammation and oxidative stress (miR-27b-3p, − 21-5p, − 122-5p, − 10a-5p, − 423-5p, and −146a-5p) were increased in plasma extracellular vesicles of HIV-positive patients, when compared to HIV-negative individuals (Chettimada et al. 2020; Meseguer-Donlo et al. 2023). Another study surmised that elevated miR-20a-3p or reduced miR-186 and miR-324-5p may lead to the downregulation of Ltbp2 (which governs adipocyte differentiation, Lamin C expression, and inflammation), in HIV associated lipodystrophy (Srinivasa et al. 2021). Such a disruption can contribute to abnormal fat distribution and inflammation, playing a role in the development of HIV-associated lipodystrophy and related metabolic disturbances. Dysregulated miRNAs were also found to serve as prognostic biomarkers by revealing both the virological and immunological status at the onset of ARV treatment and while receiving cART (Consuegra et al. 2021). Future studies can help confirm the extent of specificity of these miRNAs as potential biomarkers of metabolic disorders and clinical targets.

### What is known about the role of miRNAs in HIV/AIDS, ARVs, and obesity?

**Fig. 3:**
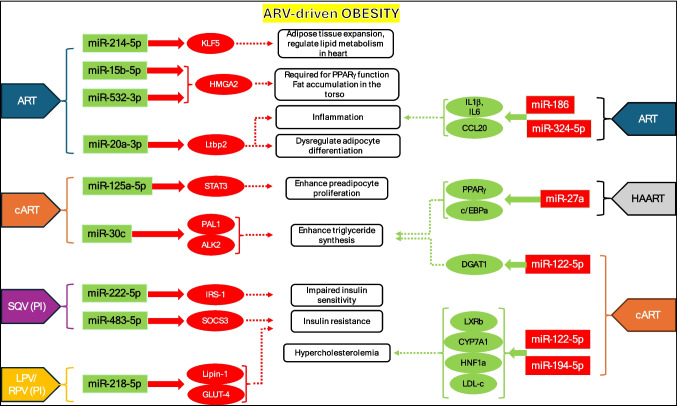
Predicted outcomes of ARV-induced obesity. ART, cART, HAART, and PI’s (Saquinavir-SQV and Lopinavir/Ritonavir-LPV/RPV) are known to induce miRNAs. These drugs may inhibit (Red box) or induce expression of miRNAs (Green box). ART is known to induce miR-214-5p, miR-15b-5p, and miR-532-3p (Bresciani et al. 2022; Squillace et al. 2014); SQV and LPV/RPV induce miR-222-5p, miR-483-5p and miR-2185p, respectively (Bresciani et al. 2019; Polus et al. 2017); cART induces miR-125a-5p and miR-30c while lowering expression of miR-122-5p and miR-194-5p (Chai et al. 2017; Kinoo et al. 2023; Squillace et al. 2014); and HAART repress expression of miR-27a (Bresciani et al. 2019). The model is based on the miRNA effects on the target molecules that are either published or predicted (KLF5) (Palioura et al. 2022) by us. The drug induced changes to miRNA > target expressions may result in beige fat accumulation (Shen et al. 2022), fat accumulation in torso (Bresciani et al. 2022), enhance preadipocyte proliferation (Xu et al. 2018) and triglyceride synthesis (Benavides-Aguilar et al. 2023; Bresciani et al. 2022; Karbiener et al. 2011), impaired insulin sensitivity (Li et al. 2020a), insulin resistance (Gallo et al. 2018), and hypercholesterolemia (Kinoo et al. 2023); all of which promote different aspects of obesity in HIV patients under ARV

Since the discovery of miRNAs in the 1990s, their role as biomarker in research has expanded significantly (Sado et al. 2024). However, few studies have explored the direct connection between the roles of miRNAs in ART-induced obesity in HIV-positive individuals. Search results using key words such as HIV, obesity, antiretrovirals, and miRNAs yielded only three published records in *Pubmed.gov*. Based on the published data on the miRNAs altered in patients on different ARV-treatments, we propose a model to explain how these select miRNAs may induce obesity (Fig. 3). This model can serve as a starting point for future studies. We propose the following three steps to understand ART-induced weight gain in terms of miRNA biology and they are as follows:

(i) Conduct a miRNA-seq study on HIV patients on different ARV treatment regimens to confirm our hypothetical model (Fig. 2) and establish a miRNA profile map at different stages of treatment.
(ii) Identify potential miRNA(s) and their target genes capable of altering weight gain pathways.
(iii) Long term benefits of the above basic science research can be used in designing and developing early-stage miRNA biomarkers and miRNA-based therapeutics to diagnose and treat ARV-driven weight gain, respectively.

Any of the miRNAs listed in Fig. 3 if induced early on by ARV in HIV patients can potentialy serve as a biomarker(s) of obesity. miRNAs are the next generation biomarkers as they can be monitored by minimal invasive procedures as obtaining saliva or body fluids (Li et al. 2025).

Obesity is a multifactorial disease (Maxim et al. 2025). ARV-induced weight gain may result from adipose tissue expansion, inflammation, fat accumulation, preadipocyte proliferation, triglyceride synthesis, insulin resistance, hypercholesterolemia, and many more unexplored factors (Fig. 3). miRNAs can directly regulate obesity by regulating adipogenesis, insulin resistance and inflammation (Heyn et al. 2020) or indirectly by altering angiogensis (Corvera et al. 2022; Urbich et al. 2012). miRNAs exhibit a range of postulated effects that are subject to modulation by a variety of factors. These factors encompass, but are not limited to, the presence of other miRNAs, specific pathologies such as virus infections, cancer, obesity, and even external variables like pharmaceutical interventions. As illustrated in Fig. 3, miRNAs may regulate ARV-induced obesity by altering expression of different targets engaged in supporting signaling critical to adipose accumulation and body weight gain. As miRNAs are regulators of gene expression (Rishik et al. 2025), they have the potential to serve as an invaluable resource in the identification of targeted therapeutics. The therapeutics may range from the use of relevant miRNA mimics, inhibitors, or miRNA precursors aimed at lowering risk of obesity. They are termed as oligonucleotide therapeutics (Roberts et al. 2020). Such synthetic oligos are able to restore upregulated/downregulated levels of intrinsic miRNAs, allowing for parallel regulation of multiple genes involved in a particular disease (Kim et al. 2022; Nappi 2024). The only caveat in the use of this approach is that the technology is still at its initial stages of development. There have been a few attempts in the form of clinical trials to test oligonucleotides as therapeutics to treat viral infection (Janssen et al. 2013) and cancer (Hong et al. 2020) but did not come to fruition primarily because of other far-reaching side effects. One of the major concern with the use of these oligonucleotide therapeutics is the stability and drug delivery that has been reinvented constantly (Dasgupta and Chatterjee 2021). Albeit, the good news is that research is never ending to developing this approach of using oligos as therapeutics: RGLS4326 (an oligo developed to inhibit miR-17) has been developed by Regulus Therapeutics to treat autosomal dominant polycystic kidney disease and is currently undergoing preclinical evaluation; and recently, a phase I study is underway to test the ability of miR-16-based microRNA mimic contained within a TargomiR (minicell) to treat patients with recurrent malignant pleural mesothelioma and non-small cell lung cancer (ClinicalTrials.gov ID NCT02369198). Future studies may use a miRNA mimic/inhibitor cocktail (Hu et al. 2011) that could alter multiple miRNA pathways and hence impeding ARV-driven obesity in HIV infected patients. An alternate approach can include developing sequence-based design of small molecules that target miRNAs (Disney et al. 2020). One major advantage of using oligo based therapeutic is in targeting several genes using a single oligo. For example, an oligo inhibiting miR-122-5p or miR-194-5p (Fig. 3) may alter functions of LXRb, CYP7A1, HNF1a, and LDL-c genes at the same time and therby lowering cholesterol levels in HIV patients on ARV.

Genetic polymorphism in HIV-infected patients significantly alter the reponses to ARV (Singh et al. 2024; Tsuchiya et al. 2025). On the same note, genetic polymorphisms in miRNA may alter miRNA expression, maturation or mRNA recognition (Gilyazova et al. 2023) and play a crucial role as a determinant of the disease progression or the manner in which HIV patient responds to an ARV regimen. miRNA polymorphisms should be taken very seriously: a polymorphism in a miRNA may result in loss or gain of function affecting expression of several genes and have serious ramifications, whereas a polymorphism in miRNA target site is more target/pathway specific (Mishra and Bertino 2009). Recent studies determined obesity-related single-nucleotide polymorphisms (SNPs) associated with weight gain in HIV-infected patients after initiating the ARV (Berenguer et al. 2023). Future studies aimed at deciphering the miRNA polymorphisms linked with ARV and obesity may help us understand the biology of miRNA in response to ARVs and aid in the development of better therapeutic options to treat obesity in HIV patients.

## Study limitations

The focus of this review was aimed to succinctly summarize the recent progress in ART-induced obesity, and the putative roles and benefits of miRNAs in regulating obesity-related genes in HIV-infected patients. Majority of this article included peer-reviewed literature and only a few clinical trials (based on what was reported/published at the time of writing this manuscript). We are sure and confident that the findings from clinical trials in the future may have relevant implications to the conclusions of this study.

## Conclusion

Past and present research has allowed for advancements and improvements in the development of ART therapy to treat HIV infections. These include simplifying the medication doses and approving new methods of treatment and prevention. Nonetheless, a major concern with these ART therapy regimens are the associated side effects. These side-effects range from obesity to decreased bone density. We propose that adopting oligonucleotide therapeutic strategies will help us in addressing these concerns. miRNAs are promising targets primarily because they are known to regulate the dogmatic gene > protein engine in a biological system. The research on miRNAs is still in its early phase. Therefore, we assert the need for continued investigation on these micro-molecules. We are currently testing dynamic changes to miRNA expression profiles in patients receiving ARVs over a specific time while simultaneously monitoring potential weight gain. Such studies will not only aid in determining biomarkers of obesity but also provide us with suitable targets that may be used to treat ARV-induced obesity. Moreover, miRNAs will have a crucial role in RNAi-based next-generation therapeutics.

## Data Availability

No datasets were generated or analysed during the current study.
